# Supplementary material for: Mechanisms governing the pioneering and redistribution capabilities of the non-classical pioneer PU.1
Source: Nat Commun. 2020 Jan 21;11:402. doi: 10.1038/s41467-019-13960-2 (PMC6972792; doi:10.1038/s41467-019-13960-2)
Supplement: Supplementary file 7 — Source data [file 41467_2019_13960_MOESM7_ESM.zip › Source_Data/Figure6/Figure6B_MotifScanOutput/ETS1_mutPU.1/homerResults/motif37.similar.html]

motif37

## Information for motif37

C
T
G
A
A
G
T
C
A
C
T
G
A
C
G
T
A
T
G
C
C
G
T
A
A
G
T
C
  
Reverse Opposite:  

C
T
A
G
A
C
G
T
A
T
C
G
C
G
T
A
A
G
T
C
A
C
T
G
A
G
C
T
  

|  |  |
| --- | --- |
| p-value: | 1e-39 |
| log p-value: | -9.066e+01 |
| Information Content per bp: | 1.873 |
| Number of Target Sequences with motif | 1130.0 |
| Percentage of Target Sequences with motif | 8.38% |
| Number of Background Sequences with motif | 1788.4 |
| Percentage of Background Sequences with motif | 5.10% |
| Average Position of motif in Targets | 104.0 +/- 55.8bp |
| Average Position of motif in Background | 99.9 +/- 64.0bp |
| Strand Bias (log2 ratio + to - strand density) | 0.0 |
| Multiplicity (# of sites on avg that occur together) | 1.17 |
| Motif File: | file (matrix) reverse opposite |

### Similar de novo motifs found

|  |  |  |  |  |  |  |  |
| --- | --- | --- | --- | --- | --- | --- | --- |
| Rank | Match Score | Redundant Motif | P-value | log P-value | % of Targets | % of Background | Motif file |
| 1 | 0.917 | G A C T T A G C T C G A A G T C C T A G A G C T A T C G C T G A G A T C | 1e-37 | -87.223352 | 5.04% | 2.60% | motif file (matrix) |
| 2 | 0.855 | C T A G A C G T A G T C C G T A A G T C C T A G A G C T A C T G | 1e-32 | -74.525595 | 3.01% | 1.32% | motif file (matrix) |
| 3 | 0.776 | A G C T A T G C G T C A G A T C T C A G A C G T A C T G C G T A A G T C A G C T A G T C A T C G T C A G T A C G | 1e-31 | -73.427996 | 1.82% | 0.60% | motif file (matrix) |
| 4 | 0.803 | G A C T A T G C T C G A A G T C T C A G C G A T T A C G C T G A A G T C T C A G | 1e-29 | -67.136802 | 3.55% | 1.77% | motif file (matrix) |
